# Genotype Distribution of *CNDP1* Polymorphisms in the Healthy Chinese Han Population: Association with HbA1c and Fasting Blood Glucose

**DOI:** 10.1155/2020/3838505

**Published:** 2020-07-18

**Authors:** Shiqi Zhang, Juan Xu, Di Cui, Shujuan Jiang, Xin Xu, Yi Zhang, Dongchun Zhu, Li Xia, Benito Yard, Yonggui Wu, Qiu Zhang

**Affiliations:** ^1^Department of Endocrinology, The First Affiliated Hospital of Anhui Medical University, Hefei 230022, China; ^2^Vth Department of Medicine (Nephrology/Endocrinology/Rheumatology), University Medical Center Mannheim, University of Heidelberg, Mannheim 68167, Germany; ^3^Department of Nephrology, The First Affiliated Hospital of Anhui Medical University, Hefei 230022, China

## Abstract

We have previously reported that the *CNDP1* (CTG)_5_ allele affords protection against diabetic nephropathy (DN) in patients with Type 2 diabetes (T2DM) of Caucasian origin. Because the incidence of ESRD attributable to both Type 1 diabetes (T1DM) and T2DM is higher among South Asian than Caucasian people, the present study assessed relevant *CNDP1* polymorphisms and their association with metabolic parameters in the Chinese Han population. To this end, the (CTG)_n_ allele distribution along with 5 relevant SNPs in the *CNDP1* gene, previously reported to be associated with DN in non (CTG)_5_ carriers of Afro-American ethnicity, were determined in 663 healthy individuals. The (CTG)_6_ homozygous genotype was the most prevalent (84.5%) genotype in the Chinese Han population. The (CTG)_5_ and (CTG)_4_ alleles were present in a small minority of individuals accounting for 15.2% and 0.3% of genotypes with at least one (CTG)_5_ or one (CTG)_4_ allele, respectively. Only 0.5% of individuals carried the homozygous (CTG)_5_ genotype and individuals carrying the homozygous (CTG)_4_ genotype were not found. The minor allele frequencies (MAFs) of the 5 SNP were 0.197 (C allele for rs4892247), 0.0855 (C allele for rs62099905), 0.085 (G allele for rs62099906), 0.066 (T allele for rs62099907), and 0.18 (A allele for rs72979715). All the SNPs except rs4892247 genotypes were in the Hardy-Weinberg equilibrium. Neither the (CTG)_n_ polymorphism nor the latter three SNPs reached significance when compared with different metabolic parameters. In contrast, individuals with the TT genotype of rs62099905 presented lower fasting blood glucose but higher HbA1c levels. In conclusion, the rs62099905 in the *CNDP1* gene is associated with serum glucose levels in the healthy Chinese Han population, while for the *CNDP1* (CTG)_n_ polymorphism, no association with serological parameters was found.

## 1. Introduction

It has been reported that patients with Type 2 diabetes (T2DM) carrying the homozygous *CNDP1* (CTG)_5_ genotype have a reduced risk to develop diabetic nephropathy (DN) as compared to T2DM patients carrying other genotypes [1]. The *CNDP1* (CTG)_n_ polymorphism is situated in the hydrophobic part of the carnosinase 1 (CN-1) signal peptide and may affect CN-1 secretion into serum. In vitro studies have suggested that the shorter (CTG)_5_ allelic variant is less efficiently secreted [2], which might explain why (CTG)_5_ homozygous individuals have lower serum CN-1 levels [1].

CN-1 is a dipeptidase which selectively hydrolyzes the histidine-containing dipeptides (HCD) carnosine, anserine, and homocarnosine. These HCD have a broad spectrum of protective effects including antioxidative and antiglycative properties which may explain their beneficial effect in the context of diabetes and other disorders related to oxidative stress [3]. Indeed, oral carnosine supplementation ameliorates DN [4] and diabetic retinopathy (DR) [5] in Type 2 and Type 1 diabetic models while overexpression of CN-1 aggravates diabetes [4]. Although the number of studies performed in humans is limited, it seems that HCD also improves obesity and glycemic outcomes in men [6, 7]. Yet, in humans, the protective effect of HCD is hampered by rapid hydrolysis of HCD because of high CN-1 activities and concentrations in human serum. Hence, carnosine can only transiently be detected in human serum, shortly after oral ingestion in individuals with low CN-1 activity [8]. Based on the foregoing, it was hypothesized that the *CNDP1* (CTG)_5_ homozygous genotype affords protection because it is associated with lower serum CN-1 activities which may facilitate higher tissue carnosine concentrations [8, 9].

The prevalence of the (CTG)_5_ and (CTG)_6_ alleles strongly varies among different ethnicities. The (CTG)_5_ allele is relatively common in Caucasians with 38.6% and 29.3% (healthy controls vs. DN-ESRD patients) of individuals being homozygous for this allele [10]. The proportion is similar in India (38.4% vs. 24.3%, healthy controls vs. DN patients) [11]. However, in the East Asian population, the prevalence of the (CTG)_5_ allele seems to be much rarer. In the Netherlands, Surinamese migrants from South Asian origin have a significantly lower frequency of the homozygous (CTG)_5_ genotype as compared to White Dutch (23% vs. 41.3%) [12]. In the Japanese population, frequencies of approximately 0.1% for this genotype are found in healthy individuals and in patients with DN [13]. Likewise, a study in peritoneal dialysis patients in Hong Kong revealed that the majority of patients (80.3%) were homozygous for the (CTG)_6_ allele, whereas the percentage of (CTG)_5_ homozygous patients was less than 1% [14]. Hence, the frequency of the protective (CTG)_5_ allele is considerably lower in the South Asian population, a population known to have a higher incidence of DN as compared to white European people [15, 16]. Apart from the (CTG)_5_ and (CTG)_6_ alleles, also the (CTG)_4_, (CTG)_7_, and (CTG)_8_ alleles have been detected in different populations, albeit at significantly lower frequencies.

In addition to the (CTG) trinucleotide repeat polymorphism, single nucleotide polymorphisms (SNPs) within the *CNDP1* gene have been postulated to affect renal complications in patients with T2DM. As such, the rs12604675 seems to be associated with overt proteinuria, but not with ESRD in Japanese women with T2DM [13]. Studies performed by McDonough pointed out that rs6566810, rs17089362 and rs6566810, rs17089362, rs890336 might mask the protection of the (CTG)_5_ allele [17].

Because current knowledge on the *CNDP1* genotypes in the Chinese population is still fragmentary, this study was conducted to delineate the distribution of *CNDP1* genotypes, with focus on relevant polymorphisms reported to affect disease course of—or susceptibility to DN and their potential association with biomarkers of renal function or other metabolic parameters in a nondiabetic Chinese population.

## 2. Materials and Methods

### 2.1. Subjects

A total of 813 subjects were recruited from the community clinics in the Xiyuan district in Hefei city (PR China). Of these subjects, 150 were excluded because of incomplete genotyping data or missing informed consent. The remaining 663 subjects were all adults of Chinese Han origin and did not fulfill one or more of the following exclusion criteria: (1) cardiac disease; (2) brain disease; (3) hepatopathy; (4) kidney disease; (5) diabetes. All subjects gave their written informed consent before recruitment. This project is approved by the local ethical committee (No. PJ2018-05-09).

### 2.2. DNA Isolation

Genomic DNA was isolated from whole blood by the Genomic DNA extraction kit (Invitrogen, USA) according to manufacturer's instruction. DNA samples were stored at -20°C until use.

### 2.3. CTG Polymorphism Genotyping

A 167 base pair fragment from exon 1 of the *CNDP1* genes, which included the (CTG)n polymorphism, was amplified by standard PCR methods using a fluorescence-labeled forward primer (5′FAM-AGGCAGCTGTGTGAGGTAAC-3′) and an unlabeled reverse primer (5′-GGGTGAGGAGAACATGCC-3′), respectively. Genotyping was performed according to fragment analysis on an ABI 3730XL (Applied Biosystems) sequencing platform.

### 2.4. SNPs Genotyping

Five SNPs within the *CNDP1* gene, i.e., rs4892247, rs62099906, rs620999905, rs62099907, and rs72979715, were selected on the basis of previous publications [13, 14, 17], provided that their minor allele frequency (MAF) in the studied Chinese Han population was more than 0.05. SNPs extension primers are shown in Table 1. All SNPs were genotyped by the SnaPshot kit (ABI) as previously described [18].

### 2.5. Statistics

Quantitative data are depicted as mean ± standard deviation. Student *t* test was used to compare differences between two groups, and One-way ANOVA was performed for more than two groups. Logarithm transformation was applied if data were not normally distributed. Correlations between categorical values were evaluated by the Pearson correlation coefficient. The continuous and categorical variables, which related to *CNDP1* genotyping with a *p* value of < 0.25 of previous univariate analysis were selected for subsequent binary or multinomial regression analysis. Significance was defined according to a *p* < 0.05. Deviations from Hardy-Weinberg equilibrium were evaluated with a Pearson's *χ*^2^ goodness-of-fit test. Statistical analyses were performed with SPSS16.0.

## 3. Results

Demographic data from the 663 included individuals are shown in Table 2. They ranged from 24 to 90 years of age, with mean values for waistline, body mass index (BMI), and blood pressure in the normal range. As expected from the inclusion criteria, there were no signs of diabetes or renal function impairment in this cohort of individuals. However, 92 out of 663 individuals were prediabetes (FBG (fasting blood glucose) 6.1-6.9 mmol/L or P2hBG (2 hours postprandial glucose) 7.8-11.0 mmol/L), 43 were obese (BMI more than 28.5 kg/m^2^); 193 individuals had hypertriglyceridemia (serum triglyceride more than 1.7 mmol/L); and 60 individuals had hypercholesterolemia.

Genotyping data for the *CNDP1* (CTG)_n_ genotype distribution was complete for all 663 individuals as depicted in Table 3. The majority of individuals (84.5%) carried the homozygous (CTG)_6_ genotype, followed by individuals that were heterozygous for the (CTG)_5_ and (CTG)_6_ allele (14.8%). Only 3 out of 663 individuals (0.5%) were homozygous for the (CTG)_5_ allele, and likewise, the (CTG)_4_ allele was found in a small minority of individuals (2 out of 663, 0.3%) and only in combination with the (CTG)_6_ allele.

Genotyping data for the 5 selected SNPs were complete in 642 individuals for rs4892247; 641 for rs62099905; 644 for rs62099906, rs62099907, and rs72979715. SNPs were selected on the basis of previous publications [13, 14, 17], showing an association with renal parameters in patients with T2DM, provided that the minor allele frequency (MAF) of the selected SNPs was more than 0.05 for the Chinese Han population in the 1000 Genomes database. The frequencies for the different loci are shown in detail in Table 4. The minor alleles and their calculated MAF as well as the MAF in the 1000 genomes database are shown in Table 4. Moreover, all SNPs except rs4892247 were in Hardy-Weinberg equilibrium (*p* > 0.05).

We next assessed if the (CTG)_n_ polymorphism or the rest four SNPs showed an association with renal function or metabolic parameters. For the former polymorphism, patients were stratified on the basis of being homozygous for the (CTG)_6_ allele (*n* = 559) vs. all other genotypes (*n* = 104). Since urinary ACR was not normally distributed in the dataset, a log transformation was performed for ACR to reach normal distribution (shown as Log ACR). In independent *t* test analysis, only 3 variables showed a *p* value less than 0.25, including systolic blood pressure (sBP), diastolic blood pressure (dBP), and skin AGEs (data not shown). These 3 variables were subsequently included in the binary logistic regression analysis but showed no significant association with the (CTG)_n_ polymorphism (data not shown).

For associations with the selected SNP the homozygous genotypes GG in rs62099906 (*n* = 4), CC in rs62099905 (*n* = 4), and AA allele in rs72979715 (*n* = 20) were excluded from the analysis due to the small sample size. Hence, independent *t* tests for the association were later performed between AA and AG genotypes in rs62099906, between CT and TT genotypes in rs62099905, between AA and AT genotypes in rs62099907, and between AG and GG genotypes in rs72979715. Variables with *p* < 0.25 were subsequently selected for binary logistic analysis.

**Figure 1:**
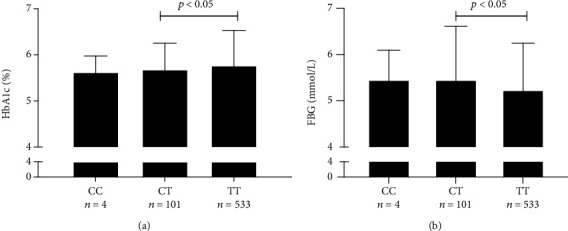
rs62099905 polymorphism in *CNDP1* gene correlated with HbA1c and fasting blood glucose levels. Legend: TT homozygosity in rs62099905 showed higher HbA1c levels (a) but lower FBG levels (b) as compared to CT heterozygosity. CC homozygosity which was not included in this statistical analysis had almost similar HbA1c and FBG levels as compared to CT heterozygosity. Abbreviations: HbA1c: Hemoglobin *β* chain (blood) -N- (1-deoxyfructose-1-yl) hemoglobin *β* chain; FBG: fasting blood glucose.

Although in univariate analysis, a number of parameters reached a threshold *p* values < 0.25, in the multivariate analysis, no significant associations for rs62099906, rs62099907, and rs72979715 were found. In contrast, rs62099905 (Table 5) showed significant associations in the multivariate model. Rs62099905 was associated HbA1c and FBG. Individuals with the TT genotype of rs62099905 displayed higher HbA1c but lower FBG levels (Figure 1, CC vs. CT vs. TT: HbA1c (%): 5.63 ± 0.35 vs. 5.68 ± 0.58 vs. 5.77 ± 0.76; FBG (mmol/L): 5.48 ± 0.61 vs. 5.48 ± 1.13 vs. 5.27 ± 0.97). The P2hBG levels between CT and TT allele were however not significant although the levels were slightly higher in the TT allele (CC vs. CT vs. TT: P2hBG (mmol/L): 6.77 ± 1.34 vs. 6.92 ± 2.87 vs. 7.01 ± 2.85).

## 4. Discussion

CN-1 gained scientific prominence by the finding that polymorphisms in the *CNDP1* gene may affect chronic kidney disease in diabetic and nondiabetic patients [1]. A (CTG)_n_ trinucleotide repeat polymorphism in the signal peptide of CN-1 has mostly been studied in this context and reported to affect CN-1 secretion [2]. The (CTG)_5_ allele seems to afford protection in patients with T2DM as the frequency of the homozygous (CTG)_5_ genotype is significantly lower in patients with DN [1]. Since the South Asian population is much more prone to develop DN than the Caucasian population [15, 16, 19], the intention of the current study was to assess the genotype distribution of polymorphisms within the *CNDP1* gene, previously reported to affect disease course of—or susceptibility to DN. We studied a healthy population to avoid a selection bias due to the high all-cause mortality in diabetic patients with impaired renal function. Notwithstanding this, we analyzed ACR and eGFR to assess for potential associations between these biomarkers and certain genotype.

The prevalence of the different *CNDP1* (CTG)_n_ alleles varies among different ethnicities. While the (CTG)_5_ and (CTG)_6_ alleles are common in the Caucasian population, the former allele is not frequently found in the Asian population [12, 14]. Our study is in line with this notion as only a remarkably low proportion (0.5%) of our studied Chinese Han population was homozygous for the (CTG)_5_ allele. This contrasted the relatively high proportion (84.5%) of individuals homozygous for the (CTG)_6_ allele, a finding that was also reported for peritoneal dialysis patients in Hong Kong [14]. Our data are also similar to that reported in the Japanese population [13]. It was found that the homozygous (CTG)_6_ genotype was most common amongst subjects followed by subjects heterozygous for the (CTG)_6_ and (CTG)_5_ allele [13]. The Hong Kong, the Japanese, and our study all three report on lower frequencies of the homozygous (CTG)_5_ as compared to the study of Mooyaart et al., in which Surinamese migrants from South Asian origin were included [12].

Neither ACR nor eGFR showed a significant relation with the *CNDP1* (CTG)_n_ genotypes in multivariant analysis. This finding is not unexpected because in a healthy population, ACR and eGFR are in a normal range, and to our knowledge, there is no direct evidence that links carnosine or CN-1 to these parameters in healthy individuals. Moreover, ACR and eGFR values were mostly below 5 mg/g, respectively, more than 60 mL/min with only small deviation as compared to diabetic patients.

The MAFs of the 5 SNPs we analyzed within the *CNDP1* gene are roughly consistent with published data, albeit that we observed a slightly lower MAF in rs4892247, rs62099906, rs62099905, and in rs62099907 and a slightly higher MAF in rs72979715. McDonough et al. reported that rs4892247 located in intron 9 of the *CNDP1* gene is associated with end-stage renal disease (ESRD) caused by diabetes [17]. Likewise, an association between chronic kidney disease and rs4892247 has been reported [20]. Unfortunately, the distribution of rs4892247 was not in Hardy-Weinberg equilibrium in our study. A larger cohort is needed to reach genetic equilibrium. For the rs62099905, significant associations were found, i.e., individuals with the TT genotype of rs62099905 presented with lower fasting blood glucose but higher HbA1c levels. This can be explained by the finding that P2hBG values contribute more to HbA1c in the Chinese population [21] and by the finding that individuals with the rs62099905 TT genotype displayed slightly higher P2hBG values. The single fasting or postprandial glucose level testing in our study, which might vary due to different factors, was also not a good predictor for HbA1c. Whether individuals carrying the TT genotypes of rs4892247 and/or rs62099905 display higher serum CN-1 activities or concentrations is currently being studied.

Although the intention of our study was to describe the genotype distribution of relevant polymorphisms within the *CNDP1* gene in the Chinese Han population, one limitation of this study is the lack of translating the individual genotypes to CN-1 concentrations or activities. In keeping with the notion that apart from the (CTG)_n_ polymorphism other factors, e.g., age and gender, are known to affect serum CN-1 concentration [22, 23], the large proportion of individuals carrying the homozygous (CTG)_6_ genotype offers the opportunity to test the influence of the other *CNDP1* polymorphisms on CN-1 concentrations. These studies are currently conducted. Further studies are also required to assess if and how serum triglycerides and HbA1c are affected by carnosine and if this correlates with *CNDP1* genotypes that are associated with high or low serum CN-1 concentrations.

In conclusion, our data confirm and extent previous studies on genotype distribution of DN relevant polymorphisms within the *CNDP1* gene. We also for the first-time report on an association between fasting blood glucose and HbA1c and the SNP within the *CNDP1* gene. Further epidemiological studies are warranted to confirm these findings, preferably also in different ethnicities.

## Figures and Tables

**Table 1:** Primers for SNPs genotyping.

	Primers	Primers for single-base extension reaction
rs4892247-F	GTGAAGGGAAAATCACAACACTTGC	TAATATAGTCTTCCATCAAGAAACTGATTA
rs4892247-R	TGCTGCGAGATACTGGGTG	
rs62099905-F	CACTCTGTGATCCTCCCACC	TAGGTATGCACCACCTGGCCTGGCTAAGTTTTAAAATCTGATTTCGCTTA
rs62099905-R	AAGATGCCCATTCCAGGTC	
rs62099906-F	GCCCAGGAATCAGAGCAGA	GGAGTTCTAGAAGAAAACAGAGGAAATGAGGCCAGGCACT
rs62099906-R	CCTCTTTCTTTGGCCTCCC	
rs62099907-F	GCCCAGGAATCAGAGCAGA	AATGAGGCCAGGCACTATGGCTCACACCTGTAATACTAGCATTTTGGGAGGCCAAAGAA
rs62099907-R	ATTTTTTTTAGAGATGGGGAC	
rs72979715-F	CACATGGGCATAGGATGGA	CACACATACACAGGGAGAACATCATAAGAAGATGAGGGCAGAGATCGGGTGATGCTTGTACAAGACAA G
rs72979715-R	CCCTACACTGGGTGGCTTA	

**Table 2:** Clinical and demographic data of all subjects.

	Female (*n* = 311)	Male (*n* = 352)
Age (year)	55.44 ± 14.39	52.39 ± 12.77
Waistline (cm)	78.75 ± 15.03	79.73 ± 22.24
Body mass index (kg/m^2^)	23.21 ± 3.59	24.58 ± 4.13
Systolic blood pressure (mmHg)	121.28 ± 20.25	126.34 ± 18.57
Diastolic blood pressure (mmHg)	76.61 ± 11.71	82.39 ± 10.87
Triglyceride (mmol/L)	1.53 ± 1.12	1.71 ± 1.30
Total cholesterol (mmol/L)	4.86 ± 0.93	4.81 ± 3.65
Serum urea nitrogen (mmol/L)	4.62 ± 1.20	4.75 ± 1.20
Creatinine (*μ*mol/L)	75.05 ± 11.27	74.86 ± 12.22
eGFR (MDRD) (mL/min)	85.75 ± 19.31	106.24 ± 28.38
Urinary ACR (mg/g)	1.74 ± 2.85	1.97 ± 5.20
Skin AGEs	70.58 ± 11.15	70.80 ± 9.75
HbA1c (%)	5.75 ± 0.56	5.76 ± 0.85
Fasting plasma glucose (mmol/L)	5.19 ± 0.85	5.41 ± 1.10
2 hours postprandial blood glucose (mmol/L)	6.94 ± 2.84	7.04 ± 2.81
Fasting C peptide (ng/mL)	1.02 ± 1.28	0.93 ± 0.70

HbA1c: Hemoglobin *β* chain (blood) -N- (1-deoxyfructose-1-yl) hemoglobin *β* chain.

**Table 3:** Distribution of *CNDP1* genotyping in China.

*CNDP1* genotyping	n	Percentage (%)
(CTG)_4-6_	2	0.3
(CTG)_5-5_	3	0.5
(CTG)_5-6_	98	14.8
(CTG)_6-6_	559	84.5
Total	663	100

**Table 4:** Minor allele frequencies and genotype distribution of *CNDP1* SNPs.

	Minor allele	MAF	MAF in 1000 genomes	SNP	*n*	Valid percent (%)
Rs4892247 genotyped *n* = 642	C	0.197	0.2787	CC	4	0.6
				TT	393	61.2
				TC	245	38.2
Rs62099906 genotyped *n* = 644	G	0.085	0.1787	AA	538	83.5
				AG	102	15.8
				GG	4	0.6
Rs62099905 genotyped *n* = 641	C	0.0855	0.1755	CC	4	0.6
				CT	102	15.9
				TT	535	83.5
Rs62099907 genotyped *n* = 644	T	0.066	0.1755	AA	559	86.8
				AT	85	13.2
Rs72979715 genotyped *n* = 644	A	0.18	0.1154	AA	20	3.1
				AG	192	29.8
				GG	432	67.1

**Table 5:** Summary of logistic regression test of variables in rs62099905 genotypes.

	Univariate analysis			Binary logistic regression analysis		
Variables	CT	TT	*p* value	*B*	OR (95% CI)	*p* value
*N* (%)	102 (15.9)	535 (83.5)	—	—	—	—
sBP (mmHg)	127.90 ± 20.65	123.45 ± 19.37	0.055∗	-0.01	0.990 (0.978-1.002)	0.096
Serum urea nitrogen (mmol/L)	4.81 ± 1.37	4.65 ± 1.16	0.249∗	-0.087	0.917 (0.760-1.106)	0.365
HbA1c (%)	5.68 ± 0.58	5.77 ± 0.76	0.186∗	0.465	1.592 (1.028-2.465)	0.037∗∗
FBG (mmol/L)	5.48 ± 1.13	5.27 ± 0.97	0.073∗	-0.276	0.759 (0.577-0.998)	0.048∗∗
Fasting C- peptide (ng/mL)	0.86 ± 0.62	0.98 ± 1.08	0.117∗	0.229	1.258 (0.886-1.784)	0.199

^∗^ *p* < 0.25; ^∗∗^*p* < 0.05. B: regression coefficient. ---: not applicable. sBP: systolic blood pressure. HbA1c: Hemoglobin *β* chain (blood) -N- (1-deoxyfructose-1-yl) hemoglobin *β* chain. FBG: fasting blood glucose.

## Data Availability

The genotyping data used to support the findings of this study are restricted by the local ethics committee in order to protect patient privacy. Data are available from Dr. Shiqi Zhang (zhangshiqi@ahmu.edu.cn), the first author of this publication, for researchers who meet the criteria for access to confidential data.

## References

[1] Janssen B., Hohenadel D., Brinkkoetter P. (2005). Carnosine as a protective factor in diabetic nephropathy: association with a leucine repeat of the carnosinase gene *CNDP1*. *Diabetes*.

[2] Riedl E., Koeppel H., Brinkkoetter P. (2007). A CTG polymorphism in the CNDP1 gene determines the secretion of serum carnosinase in Cos-7 transfected cells. *Diabetes*.

[3] Boldyrev A. A., Aldini G., Derave W. (2013). Physiology and pathophysiology of carnosine. *Physiological Reviews*.

[4] Sauerhöfer S., Yuan G., Braun G. S. (2007). L-carnosine, a substrate of carnosinase-1, influences glucose metabolism. *Diabetes*.

[5] Pfister F., Riedl E., Wang Q. (2011). Oral carnosine supplementation prevents vascular damage in experimental diabetic retinopathy. *Cellular Physiology and Biochemistry*.

[6] Menon K., Marquina C., Liew D., Mousa A., de Courten B. (2020). Histidine-containing dipeptides reduce central obesity and improve glycaemic outcomes: a systematic review and meta-analysis of randomized controlled trials. *Obesity Reviews*.

[7] Karkabounas S., Papadopoulos N., Anastasiadou C. (2018). Effects of *α*-lipoic acid, carnosine, and thiamine supplementation in obese patients with type 2 diabetes mellitus: a randomized, Double-Blind Study. *Journal of Medicinal Food*.

[8] Everaert I., Taes Y., de Heer E. (2012). Low plasma carnosinase activity promotes carnosinemia after carnosine ingestion in humans. *American Journal of Physiology-Renal Physiology*.

[9] Everaert I., He J., Hanssens M. (2020). Carnosinase-1 overexpression, but not aerobic exercise training, affects the development of diabetic nephropathy in BTBRob/obmice. *American Journal of Physiology-Renal Physiology*.

[10] Freedman B. I., Hicks P. J., Sale M. M. (2007). A leucine repeat in the carnosinase gene *CNDP1* is associated with diabetic end-stage renal disease in European Americans. *Nephrology, Dialysis, Transplantation*.

[11] Yadav A. K., Sinha N., Kumar V., Bhansali A., Dutta P., Jha V. (2016). Association of CTG repeat polymorphism in carnosine dipeptidase 1 (*CNDP1*) gene with diabetic nephropathy in north Indians. *The Indian Journal of Medical Research*.

[12] Mooyaart A. L., van Valkengoed I. G. M., Shaw P. K. C. (2009). Lower frequency of the 5/5 homozygous *CNDP1* genotype in South Asian Surinamese. *Diabetes Research and Clinical Practice*.

[13] Kurashige M., Imamura M., Araki S. I. (2013). The influence of a single nucleotide polymorphism within *CNDP1* on susceptibility to diabetic nephropathy in Japanese women with type 2 diabetes. *PLoS One*.

[14] Poon P. Y. K., Szeto C. C., Kwan B. C. H., Chow K. M., Li P. K. T. (2010). Relationship between carnosinase gene CNDP1 leucine repeat polymorphism and the clinical outcome of Chinese PD patients. *Clinical Nephrology*.

[15] Burden A. C., McNally P. C., Feehally J., Walls J. (1992). Increased incidence of end-stage renal failure secondary to diabetes mellitus in Asian ethnic groups in the United Kingdom. *Diabetic Medicine*.

[16] Lopes A. A. (2009). End-stage renal disease due to diabetes in racial/ethnic minorities and disadvantaged populations. *Ethnicity & Disease*.

[17] McDonough C. W., Hicks P. J., Lu L., Langefeld C. D., Freedman B. I., Bowden D. W. (2009). The influence of carnosinase gene polymorphisms on diabetic nephropathy risk in African-Americans. *Human Genetics*.

[18] Bujalkova M., Zavodna K., Krivulcik T. (2008). Multiplex SNaPshot genotyping for detecting loss of heterozygosity in the mismatch-repair genes *MLH1* and *MSH2* in microsatellite-unstable tumors. *Clinical Chemistry*.

[19] Chandie Shaw P. K., Baboe F., van Es L. A. (2006). South-Asian type 2 diabetic patients have higher incidence and faster progression of renal disease compared with Dutch-European diabetic patients. *Diabetes Care*.

[20] Guo J., Chen L. M., Chang B. C., Zheng M. Y., Wen J. J. (2016). Association analysis of polymorphism in CNDP1 gene and chronic kidney disease in type 2 diabetic Chinese Han population. *Chinese Journal of Diabetes*.

[21] Yan R., Hu Y., Li F. (2019). Contributions of fasting and postprandial glucose concentrations to haemoglobin A1c in drug-naïve mal-glucose metabolism in Chinese population using continuous glucose monitoring system. *International Journal of Endocrinology*.

[22] Mooyaart A. L., Zutinic A., Bakker S. J. L. (2010). Association between *CNDP1* genotype and diabetic nephropathy is sex specific. *Diabetes*.

[23] Lenney J. F., George R. P., Weiss A. M., Kucera C. M., Chan P. W. H., Rinzler G. S. (1982). Human serum carnosinase: characterization, distinction from cellular carnosinase, and activation by cadmium. *Clinica Chimica Acta*.

